# DNA METHYLATION: CAUSE OR CONSEQUENCE OF AGING?

**DOI:** 10.1093/geroni/igz038.127

**Published:** 2019-11-08

**Authors:** Morgan E Levine, Sara Hagg

**Affiliations:** 1 Yale University School of Medicine, New Haven, Connecticut, United States; 2 Karolinska Institutet, Stockholm, Sweden

## Abstract

Epigenetic changes are one of the Hallmarks of Aging. DNA methylation is a key epigenetic mark that has been shown to change during aging. Several "clocks" have been developed whereby changes in DNA methylation can be used to predict chronological, and perhaps, biological age. This symposium will focus on recent advances in understanding how and why changes in DNA methylation occur during aging and whether these changes play a causal role in age-related functional declines and disease.

